# Calcium Signaling and Neurodegeneration

**Published:** 2010-04

**Authors:** I.B. Bezprozvanny

**Affiliations:** University of Texas Southwestern Medical Center; Institute of Cytology, Russian Academy of Sciences

**Keywords:** Alzheimer’s disease, Parkinson’s disease (PD), amyotrophic lateral sclerosis, Huntington’s disease, spinocerbellar ataxias, calcium channels, calcium signaling, mitochondria, transgenic mice, clinical trials, imaging, memantine, dimebon, riluzole

## Abstract

Neurodegenerative disorders, such as Alzheimer’s disease (AD), Parkinson’s disease (PD), amyotrophic lateral sclerosis (ALS), Huntington’s disease (HD), and spinocerebellar ataxias (SCA) are very important both for fundamental science and for practical medicine. Despite extensive research into the causes of these diseases, clinical researchers have had very limited progress and, as of now, there is still no cure for any of these diseases. One of the main obstacles in the way of creating treatments for these disorders is the fact that their etiology and pathophysiology still remain unclear. This paper reviews results that support the so–called “calcium hypothesis of neurodegenerative diseases.” The calcium hypothesis states that the atrophic and degenerative processes in the neurons of AD, PD, ALS, HD, and SCA patients are accompanied by alterations in calcium homeostasis. Moreover, the calcium hypothesis states that this deregulation of calcium signaling is one of the early–stage and key processes in the pathogenesis of these diseases. Based on the results we reviewed, we conclude that the calcium channels and other proteins involved in the neuronal calcium signaling system are potential drug targets for AD, PD, ALS, HD, and SCA therapy.

## Neuronal Ca^2^^+^ Signaling

Calcium signaling in neurons connects membrane excitability with the biological function of the cell [1]. Since Са^2+^ channels are located on the boundary between the “electrical” and the “signaling” worlds, they play a key role in various aspects of the neuronal function. Ca^2+^ signaling is required for short–term and long–term synaptic plasticity. Because of its extreme importance, neurons use multiple mechanisms to control intracellular levels of Ca^2+^, usually within local signaling microdomains.

**Table 1:** Neurodegenerative diseases (Bezprozvanny, 2009)

Disease	Affected neurons	Age of onset	Sporadic/inherited	Genes	Drugs	Target	Effect
AD		Neurons of the cortex and hippocampus		>65		95% sporadic, 5% inherited		APP PSEN1 PSEN2		Namenda (Memantine)	Blocks NMDA-receptors, reduces toxicity	Moderately improves cognitive function
Donepezil (Aricept), Galantamine (Razadyne), Rivastigmine (Exelon)	Inhibitors of acetylcholine esterase. Increase the amount of acetylcholine in the brain	Moderately improves cognitive function
PD	Dopaminergic neurons of the pars compacta of the substantia nigra	>65	95% sporadic, 5% inherited	Synucl, LRRK2, Parkin, PINK1, DJ-1	L-Dopa (Levodopa)	Increases the amount of dopamine in the neurons of the substantia nigra	Symptomatic relief
ALS	Motor neurons	40–60	95% sporadic, 5% inherited	SOD1	Riluzole (Rilutek)	Antiglutamate effect (activates the capture of glutamate, blocks the NMDAR receptor and Na-channels)	Increases life-span by a few months
HD	Medium spiny neurons of the striatum	40–50	100% inherited	Huntingtin	Tetrabenzine (Xenazine)	Antidopamine effect (inhibits VMAT, lowers the amount of excreted dopamine)	Reduces chorea
SCA	Various regions of the brain involved in motor control	40–50	100% inherited	Ataxins	None	None	None

A variety of Ca^2+^ channels are involved in neuronal Ca^2+^ signaling: the voltage–dependant Ca^2+^ channels of the plasma membrane (VGCC ), NMDA receptors, AMPA receptors, TRP channels, and depot–controlled channels. Release of Ca^2+^ from the intracellular ER depot is mediated by inositol–1,4,5–triphosphate receptors (InsP_3_R) and ryanodine receptors (RyanR). The SERCA pump in the ER, the Ca^2+^ pump of the plasma membrane, and the Na^+^/Ca^2+^ exchanger of the plasma membrane are involved in the accurate control of the Ca^2+^ level in the cytosol in a very narrow range. The mitochondria play a very important role in the formation of cytosolic Са^2+^ signals. The mitochondrial Ca^2+^ uniporter (MCU) is an ion channel involved in the rapid and massive entrance of calcium into the mitochondria. A large number of Ca^2+^–binding proteins are involved in maintaining a certain level of Ca^2+^ in the cytosol (such as calbindin–D28, calretinin, and parvalbumin) and inside the ER (such as calreticulin and calnexin) in neurons.

Since neurons are highly sensitive to changes in the intracellular Ca^2+^ concentration, they have a whole range of Ca^2+^–dependent structures, including proteins that are involved in the fusion of synaptic vesicles with the presynaptic membrane (such as synaptotagmins), Ca^2+^–dependant kinases and phosphatases (such as the Ca^2+^/CaM kinase and the Ca^2+^–dependant phosphatase calcineurin), Ca^2+^–dependent signaling enzymes (such as Ca^2+^–dependent adenylate cyclase and Ca^2+^–dependent NO–synthase), and Ca^2+^–dependent transcription factors (such as the cAMP–dependent element–binding protein, calcineurin B–controlled activated T–lymphocyte nuclear factor, and Ca^2+^ binding downstream regulatory element modulator). Such a variety of Ca^2+^–dependent elements makes the fine Ca^2+^–dependent regulation of a neural function on time–scales possible in the microsecond range (as is the case of the Сa^2+^–dependent fusion of a synaptic vesicle with the presynaptic membrane), in second and minute ranges (as in the case of Ca^2+^–dependent phosphorylation and dephosphorylation), and up to day and year ranges (for Ca^2+^–dependent changes in neural gene expression). These Ca^2+^–dependent processes lead to short– and long–term changes in neuronal excitability (by changing the activity of ion channels and the expression pattern) and changes in synaptic transduction (by modifying the synaptic machinery and forming or disjoining synaptic connections). Since neurons are extremely sensitive to changes in Ca^2+^ signaling, even fine defects and deregulation of Ca^2+^ signaling can have destructive consequences [2].

## Ca^2+^ Blockers and a Complex Approach for Treating Neurodegenerative Disorders

Neurodenerative disorders, such as Alzheimer’s disease (AD), Parkinson’s disease (PD), amyotrophic lateral sclerosis (ALS), Huntington’s disease (HD), and spinocerbellar ataxias (SCA), are a very important problem both for fundamental science and for practical medicine. Despite extensive research into the causes of these diseases, clinical researchers have had very limited progress and as of now there is still no cure for any of these diseases. Therapeutic drugs used for treating these disorders have only a limited effect, causing only temporary relief of the symptoms or slowing the disease’s progression (Table 1). A significant advance in the study of these disorders was achieved with the discovery of mutations that cause the pathological processes. HD and SCA are genetic disorders, and the genes which cause these diseases were cloned around 15 years ago (Table 1). Most cases of HD, PD, and ALS are sporadic, but around 5% of the patients inherit the disorder. Most of the genes which are involved in the development of the heritable form of the disease have been cloned (Table 1). The study of the genes which cause the above–mentioned diseases allowed researchers to form a mechanistic hypothesis for the pathological process and creates a mouse model for studying these pathologies. Most attempts at studying the above–mentioned pathologies are focused on identifying the main causes of diseases and developing approaches to affect these causes. For instance, the main cause of AD was thought to be the formation of amyloid. Because of this, the main research efforts are directed at preventing the accumulation of amyloid by blocking its production or facilitating its clearance from the brain. In case of HD, the main reason is the expression of a mutant form of the huntingtin (Htt) protein. This means that most experimental efforts are directed at lowering mutant Htt expression in the brain (such as by using interfering RNA or a antisense knockdown).

Despite impressive scientific results, these approaches are hard to use in clinical treatment. For instance, in case of AD, the clinical trials of the amyloid–binding drug trampiprosate (Alzhemed) and the γ–secretase inhibitor tarenflurbil (Flurizan) were both unsuccessful. Clinical trials of amyloid–binding monoclonal antibodies (Bapineuzumab) had a very limited or even a negligible effect. The clinical trials of approaches for treating HD have problems with devising an adequate approach for siRNA or antisense RNA delivery into the human brain. There is still no solution for this problem, and clinical trials cannot be performed. While the focused attention on amyloid and mutant Htt is understandable, it is worth mentioning that the collected data indicate that these are targets which are very difficult to affect and that the creation of successful therapy based on these approaches will take a long time and take up considerable resources. In addition to developing methods for treatment, we propose a treatment that can delay the age at which the symptoms of the disease are manifested and/or lower the degree of the disease manifestation. Further, we will focus attention on the concept that the proteins involved in the calcium signaling in neurons are promising targets for developing “disease–delaying” therapy for neurodegenerative pathologies. We surmise that the most promising approach for clinical treatment will be a combination of approaches developed for each disease (such as amyloid–directed therapy for AD and huntingtin–directed therapy for HD) and of Ca^2+^ blockers.

## Neuronal Ca^2^^+^ Signaling and Aging

Our neurons are the same age as us. Thus, it is not surprising that the risk of neurodegenerative diseases increases with age (Table 1). Comparative studies of neurons from young and old rodents have shown that the neuronal Са^2+^–signaling system experiences changes during aging. These data have been extensively published in the scientific press [2]. Recently, an integral model of age–dependent changes in hypocampal Ca^2+^ signaling has been proposed [3]. One of the main features of aging neurons is an increase in the Ca^2+^ concentration via active Ca^2+^ release from the intracellular depot using InsP_3_R and RyanR, an increased release of Ca^2+^ through the L–type VGCC, an increase of the slow trace hyperpolarization caused by the activation of Ca^2+^–dependent K+ channels, a lowered involvement of NMDAR–mediated Ca^2+^ entrance, and a lowered buffer capacity of the cytosol and activation of calcineurin and calpains. Such changes in the neuronal Ca^2+^ dynamics lead to increased sensitivity, to the induction of long–term depression, and to the increased threshold frequency required for long–term potentiation in aging neurons [4]. The importance of these changes was also discussed in connection with the age–dependent disorders of the memory function [4].

The mechanisms involved in age–dependent changes in neuronal Ca^2+^ signaling have still not been elucidated. One possible scenario is connected with the age–dependent defects of mitochondrial function caused by the overall oxidative damage sustained by the mitochondria. The mitochondria of aged neurons are depolarized and less effective in the control of Ca^2+^ uptake [2]. Age–dependent changes in the transcription of Ca^2+^–signaling proteins were also discovered [2]. Some of these changes were directly dependent on the aging process and some of them were compensatory, but the whole picture is in agreement with the presence of age–dependent changes in the neuronal Са^2+^–signaling system on various levels.

## Neuronal Ca^2+^ Signaling and Huntington’s Disease

**Fig. 1:**
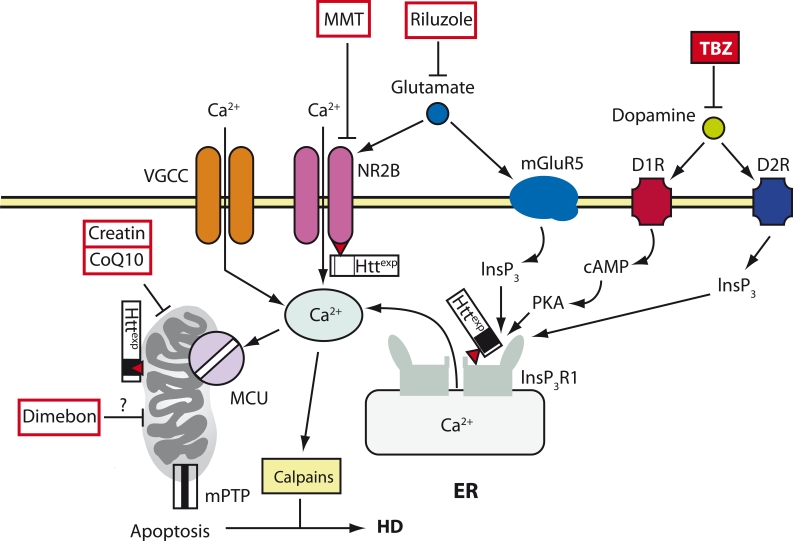
A model of Ca^2+^ deregulation during HD (cited from (Tang et al., 2007)). In MSNs during HD, Httexp disrupts Ca^2+^ signaling by three syn- ergistic mechanisms. Httexp increases the function of the NR2B-bearing NMDA receptor (probably by increasing its transport into the plasma membrane). Httexp tightly binds to the С-terminus of InsP3R1 and increases its affinity to InsP3. The low level of glutamate secreted by the neurons of the corticostriatal projection causes an excessive influx of Ca^2+^ via the NMDA receptor and the release of Ca^2+^ from the ER via InsP3R1. The additional uptake of Ca^2+^ into MSNs is mediated by VGCC. Dopamine excreted by the dopaminergic neurons of the mesencephalon stimulates the type-1 (D1R) and type-2 (D2R) dopamine receptors, which are highly expressed in MSN. D1R is connected with an adenylate cyclase, and together they increase the cAMP level and activate the protein kinase A (PKA). PKA enhances the glutamate-induced Ca^2+^ signals by increasing the activity of the NMDA receptor and InsP3R1. D2R is directly involved in the produc- tion of InsP3 and the activation of InsP3R1. The excessive uptake of Ca^2+^ activates calpain, which cleaves Httexp and other substrates. The excess of Ca^2+^ in the cytosol leads to the capture of Ca^2+^ by the mitochondria via MCU, which in turn induces the opening of mPTP and apoptosis. The calcium regulation of mitochondria is also disrupted due to the direct interaction between Httexp and the mitochondria. The antidopamine drug tetrabenzine (TBZ) has been approved in the United States for the symptomatic treatment of HD. The NMDA receptor antagonist memantin (MMT), the soluble “mitochondrial agent” dimebon and “mitochondrial stabilizers” creatin and coenzyme Q10 (CoQ10) are all in clinical trials. The antiglutamate agent Riluzole has passed clinical trials, but it turned out to be ineffective for HD treatment [19]

Huntigton’s disease (HD) is a genetic disorder which is caused by a single mutation: the expansion of the CAG (polyglutamine) repeat in the huntingtin (Htt) gene [5] (Table 1). Medium spiny neurons (MSN) in the striatum are cells that sustain the most damage during HD. Most researchers agree that the mutant protein Htt^exp^ experiences a “gain of its toxic function” [6]. The destabilization of neuronal Ca^2+^ signaling is one of the toxic functions of the Htt^exp^ protein. Studies of HD patient’s brains and also model experiments with mice show that the brain experiences sequential changes in the expression levels of Ca^2+^–signaling proteins [7]. We proposed the “calcium hypothesis for HD” [8]. There are several main pathways for the effect of Htt^exp^ on Ca^2+^ signaling in MSN (Fig. 1). Our laboratory has established that Htt^exp^ directly and specifically binds the C–terminus of InsP_3_R1 [9]. The association between Htt^exp^ and InsP_3_R1 was independently discovered by random screening [10]. Binding with Htt^exp^ increases the affinity of InsP_3_R1 for InsP_3_ [9]. The key role of InsP_3_R1 activation in Htt^exp^ neurotoxicity was confirmed experimentally in mouse MSN cultures, which were used to model HD [11, 12], and in genetic experiments on the Drosophila based HD model [10]. Recent studies show that the viral delivery of a peptide that destabilizes the interaction between Htt^exp^ and InsP_3_R1 has a protective effect on the striatum MSN in the mouse HD model both in *in vitro* and *in vivo* conditions [13]. These data confirm the importance of increased InsP_3_R1 activity in HD pathogenesis. The expression of Htt^exp^ causes increased activity of the NR 2B–bearing NMDA–receptor [14]. The increased flow through the NMDA–receptor is a consequence of the effect of Htt^exp^ on the transport of the NMDA–receptor to the plasma membrane [15]. Striatum MSNs expressing Htt^exp^ are sensitive to NMDAR–mediated toxicity. The pharmacological inhibition of the NMDA–receptor has a neuroprotective effect on a mouse MSN HD–model culture [11, 16]. Both memantin and riluzole had a neuroprotective effect on MSN cultures with HD. Memantin was more effective [17]. Memantin also had some positive effects in a small–scale experimental survey of this drug on HD patients [18], and it will soon be in the fourth phase of clinical trials for HD therapy (Table 2). Riluzole has completed the third stage of clinical trials on HD patients, but this study did not turn out to be successful [19] (Table 2).

In addition to InsP_3_R1 and to the NMDA–receptor, Htt^exp^ can also affect potential–dependent Ca^2+^ channels (VGCC). Huntingtin directly binds the α2/δ accessory subunit of VGCC [10] and the CaV2.2 pore–forming subunit of N–type VGCC [20]. The genetic removal of Dmca1D (pore–forming subunit of the L–type calcium channel in Drosophila) decreases the neurodegeneration of the photoreceptor in HD–model fruit flies [21]. An electrophysiological analysis of the striatum neurons of HD–model mice showed an initial increase of the VGCC channel density, which was followed by a decrease in their density [22]. Just as for other neurodegenerative disorders, Ca^2+^ toxicity mechanisms during HD are most often mediated by calpain activation and Ca^2+^ accumulation in the mitochondria ( Fig. 1 ). The activation of calpains is observed during HD, and calapin–mediated cleavage of Htt^exp^ and the NMDA–receptor plays a key role in the pathology of this disease [23–25]. A large body of evidence indicates mitochondrial dysfunction during HD [26]. Mitochondria isolated from the HD patient’s lymphoblasts and from the brains of transgenic HD mice exhibited clear defects of the calcium system regulation [27]. The mitochondrial function was also disrupted in cell HD models [11, 12, 16, 28]. In addition to the effect on the mitochondria caused by the excessive concentration of Ca^2+^ in the cytosol, Htt^exp^ can also affect these organelles by directly binding with their outer membrane [27] (Fig. 1). It is worth noting that clinically adequate inhibitors of mitochondrial membrane permeability demonstrated a neuroprotective effect both on cellular HD models and on animal models of this disease [11, 28].

The first drug approved for HD treatment in the United States in 2008 was a dopamine tetrabenzine antagonist (TBZ) (Table 1). TBZ is a powerful inhibitor of the monoamine vesicular transporter, which causes the depletion of the dopamine contents of presynaptic vesicles. The clinical trials demonstrated that TBZ had a reliable suppressor effect on chorea symptoms in HD patients [29]. Our laboratory studied the effects of TBZ on the mouse HD model. It was shown that the effect of this drug lowered the deficit of motor coordination in the early stages of the disease and protected the striatum neurons from degeneration under *in vivo * conditions [30]. It was concluded that dopamine and glutamate have a synergetic activity in the formation of Ca^2+^ signals in the neurons of the striatum and that the effect of TBZ might be due to lowered Ca^2+^ signaling [30] (Fig. 1). These facts confirm that TBZ cannot only be used as a drug for symptomatic treatment on late stages of the disease, but also as a drug for treating the disease presymptomatically. However, TBZ caused strong depression in some patients [29], which is why alternative dopamine antagonists should be researched, for instance, the dopamine–specific inhibitor of the vesicular monoamine transporter or blockers of D1 or D2 receptors.

**Table 2:** The most recent clinical trials of Ca^2+^ inhibitors and mitochondrial stabilizers as treatments for neurodegenerative disorders.

Disorder	Drug	Target	Clinical trial stage	Clinical trial ID	Information was supplied by	Status/commentary
AD	Dimebon	mitochondrion (?)	Phase III	NCT00675623	Medivation	Completed, unsuccessful
	Ketasyn (AC-1202)	mitochondrion	Phase II	NCT00142805	NIA	Completed
	MEM-1003	L-type VGCC	Phase II	NCT00257673	NCT00257673	NCT00257673	Memory Pharmaceuticals	Completed
	EVT-101	NR 2B NMDA-receptor	Phase I	NCT00526968	Evotec Neurosciences	Completed, Phase II is planned
HD	Dimebon	mitochondrion (?)	Phase II	NCT00497159	Medivation	Completed, weak effect on cognitive function
	Creatine	mitochondrion	Phase III	NCT00712426	MGH	Forming test subject group
	Coenzyme Q10 (CoQ10)	mitochondrion	Phase III	NCT00608881	NINDS	Forming test subject group
	Memantine	NMDA-receptor	Phase IV	NCT00652457	UCSD	Forming test subject group
	Riluzole	antiglutamate	Phase III	NCT00277602	Sanofi-Aventis	Completed, unsuccessful ([19], 2007)

## Neuronal Ca^2+^ Signaling and Spinocerbellar Ataxias

Like in the case of HD, spinocerbellar ataxias (SCA) are autosomal dominant genetic disorders caused by the expansion of the polyglutamine sequence in ataxin proteins (Atx) [5]. There is a number of observations which indicate that disorders in the neuronal Ca^2+^ signaling can play a role in the pathogenesis of these diseases. Some of these data are presented further.

SCA1 leads to the degeneration of Purkinje cells of the cerebellum caused by the expansion of CAG repeats in the cytosol/nuclear protein ataxin–1 [5]. Purkinje cells of the cerebellum express very high levels of Ca^2+^–signaling proteins and Ca^2+^–binding proteins. A decrease in the Ca^2+^–binding proteins in Purkinje cells was also observed in patients with early–stage SCA1 and in mouse models of this disease [31]. Crossing transgenic SCA1 mice with calbindin knockout mice led to an increased disease phenotype [31]. The transgenic CMA1 mouse model made it possible to observe the lowered expression of Ca^2+^–signaling proteins such as InsP_3_R1, Ca^2+^–channel TR PС3, and the ER pump SERCA2 during the early stages of the disease [32]. Albeit indirectly, these data confirm the fact that the disruption of the calcium signaling in Purkinje cells probably plays a key role in the etiology of SCA1.

During SCA2, the Purkinje cells of the cerebellum experience degeneration due to the expansion of CAG repeats in the cytosolic protein ataxin–2 [5]. The genetic connection between the polymorphism of the type–P/Q VGCC sequence and the age at which the first symptoms of SCA2 are manifested confirms the fact that Ca^2+^ signaling plays a very important role in the pathogenesis of this disease [33]. Our laboratory has discovered that the mutant form of ataxin–2 specifically binds and activates InsP_3_R1 similarly to the mutant form of Htt (article in print). We also demonstrated that inhibitors of Ca^2+^ signaling protected Purkinje cells from apoptosis during SCA2 under *in vitro* conditions and had a pronounced positive effect in experiments on transgenic mice (article in print).

During SCA3, neurons of the * substantia nigra * and the pontine nuclei experience degeneration as a result of the CAG repeat expansion in the ataxin–3 cytosolic protein [5]. Calpain–mediated cleavage of ataxin–3 plays an important role in the pathogenesis of SCA3 [34]. Recently, we showed that the mutant form of ataxin–3 specifically binds and activates InsP_3_R1 similarly to how it binds the mutant form of the Htt protein [35]. It was further determined that the long–term feeding of CMA3 mice with a RyanR inhibitor and the Ca^2+^ stabilizer dantrolen facilitated the age–dependent deficit of motor coordination in these mice and prevents the loss of neurons in the * substantia nigra* and the pontine nuclei [35].

SCA6 causes Purkinje cells of the cerebellum to degenerate as a result of the expansion of CAG repeats in the C–terminus of the CaV2.1 (the pore–forming subunit of the P/Q–type Ca^2+^ channel) [5]. It was reported that this mutation increased the activity of the P/Q–type Ca^2+^ channel in an expression system [36]. However, most recent studies of SCA6 mice have shown that this pathology is also associated with the aggregation of CaV2.1 subunits and with the reduced density of the Ca^2+^ flow through the P/Q–type channels in dendrites [37]. Thus, the issue of the precise role of Ca^2+^–signaling deregulation during SCA6 still remains unresolved. Anomalous neuronal Ca^2+^ signaling is not limited to ataxias with expanded polyglutamine repeats, but it can also play an important role in the ataxias of other types. The most recent genetic studies have shown that the cause of SCA15 is the loss of a fragment of the InsP_3_R1 gene [38].

## Neuronal Ca^2+^ Signaling and Alzheimer’s Disease

Alzheimer’s disease (AD) is a neurodegenerative disorder which causes memory loss. In most cases, AD appears sporadically and the first symptoms emerge in the elderly (after 60). A small fraction of cases (heritable AD (HAD)) are characterized by the early onset of symptoms and genetic inheritance.

## Neuronal Ca^2+^ Signaling and Sporadic AD

Sporadic AD is a “multitraget” disorder caused by the synergistic effect of several pathological factors. One of these factors is aging. The other factors are determined by the populations of neurons affected by the disease, in this case the cortical and hypocampal neurons. The main “disease–specific” factor during AD is probably the accumulation of amyloid. Since AD is a multitarget disease, the successful therapy must have a complex nature. The population of neurons which express high levels of Ca^2+^–binding proteins remain mostly untouched by AD, while the populations of neurons which express these proteins at a low level experience extensive damage. A decreased level of Ca^2+^–binding proteins is one of the most usual consequences of the natural aging process. Most likely one of the causes of an increased susceptibility of aged neurons to AD is the decreased buffer capacity of the neuronal cytosol for Ca^2+^. Neurons of elderly patients who suffer from the sporadic form of AD exhibit an activation of Ca^2+^–dependent proteases of the calpain family. Calpain activation takes place as a response to the increased levels of Ca^2+^ in the cytosol. Activated calpains cleave various proteins which are required for the normal functioning of the neuron, which results in neuronal dysfunction and apoptosis.

The mitochondria in neurons of AD patients experience severe damage. These organelles are partly depolarized, they exhibit lowered ability to bind Ca^2+^, the disruption of the stoichiometry of the electron transfer chain, and the mutation of the mitochondrial DNA. Similar – but less visible – changes also take place in the mitochondria during the natural aging process. Damage to the mitochondria is probably caused by an oversaturation of this organelle by Ca^2+^, which causes the formation of large quantities of active forms of oxygen, which then cause extensive oxidative damage to the mitochondrial DNA. Thus, mitochondria seem to be the final step in the calcium–signaling chain of this pathogenic cascade. However, it is still expected that “mitochondrial stabilizers” (such as coenzyme Q10 and creatine) should have some positive effect on these disorders. Drugs which are targeted at the mitochondrial permeability transition pore (mPTP) should be extremely useful as a “last line of defence” for the neuron delaying of the onset of neuronal dysfunction and cell death.

The aging process affects neuronal Ca^2+^ signaling and seems to be one of the factors involved in pathogenesis during sporadic AD. Thus, it is expected that blockers of Ca^2+^ signaling can have a positive effect on this disease. The NMDA–receptor antagonist Memantin has demonstrated a certain degree of clinical efficiency in AD treatment. The treatment of this disease requires the development and clinical trial of new Ca^2+^–signaling blockers by themselves and as part of a complex therapy in combination with mitochondrial stabilizers and with mPTP inhibitors.

## Neuronal Signaling and HAD

**The calcium hypothesis of AD pathogenesis. **The central idea for explaining AD pathology is currently the amyloid hypothesis, which states that the main reason for neuron death and the decreased number of synapses during this disorder is the increased production of Aβ42 amyloid peptide (or the increased Aβ42/40 ratio) [39].

**Fig. 2:**
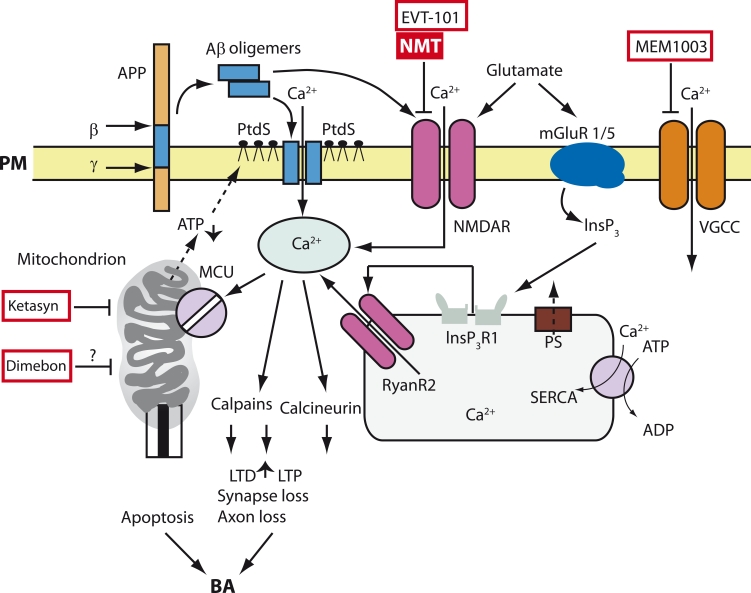
Model of Ca^2+^ deregulation in AD (Bezprozvanny and Mattson, 2008). Sequential cleavage of the β-amyloid precursor-protein (β) by the γ-secretase (γ) leads to the formation of Aβ. Aβ forms oligomers, which can integrate into the plasma membrane (PM) and form pores permeable to Ca^2+^-ions. The association of Aβ oligomers with the plasma membrane is facilitated by binding with the surface phosphatidylserine (PtdS); the aging process and Ca^2+^-mediated damage of mitochondria cause a decrease in the ATP level and lead to the transfer of PtdS from the inner to the outer surface of the plasma membrane. The decrease of the ATP level and the loss of membrane integrity cause membrane depolarization, which in turn causes an increase in the uptake of Ca^2+^ through NMDA receptors and VGCC. Aβ oligomers can also directly influence the affinity of the NMDA, AMPA, and VGCC receptors. Gluta- mate activates mGluR1/5 receptors, increases the production of InsP3, and facilitates InsP3-mediated release of Ca^2+^ from the ER. Presenilins (PS) function as channels for Ca^2+^ drain from the ER and various muta- tions associated with HAD disrupt the Ca^2+^ drain function of presenilins. This causes the excessive accumulation of Ca^2+^ in the ER. An increase in Ca^2+^ levels in the ER enhances the release of Ca^2+^ via the type-I InsP3 (InsP3R1) and type-2 ryanodine receptor (RyanR2). PS can also directly modulate the activity of InsP3R, RyanR, and the SERCA pump. The increase in cytosolic Ca^2+^ concentration activates calcineurin and calpains, which in turn enhances long-term depression (LTD), suppresses long-term potentiation (LTP), and causes the modification of the neuronal cytoskeleton and the loss of synapses and axon atrophy. The excessive amount of Ca^2+^ in the mitochondria, which appears due to the activity of mitochondrial calcium uniporter (MCU), leads to the opening of the mitochondrial permeable transit pore (mPTP) and apoptosis. An inhibitor of the NMDA receptor called memantine (MMT) has been approved for AD treatment, and a NR2B-specific antagonist EVT-101 has also been developed. Currently, several other drugs are under clinical trials for the treatment of AD: a “CNS optimized” L-type VGCC inhibitor MEM-1003, the soluble “mitochondrial agent” Dimebon, and “mitochondrial antide- pressant ” Ketasyn

Experimental proof of the amyloid hypothesis is based on the following facts: (1) amyloid plaques are accumulated in the brains of AD patients; (2) the heritable form of AD (HAD) is caused by nonsense–mutations in the β–amyloid Aβ precursor protein (АРР); and (3) HAD is also caused by nonsense mutations in presenilins, which form the catalytic subunit of γ–secretase, an enzyme that cleaves APP. Currently, amyloid–directed therapy is the central strategy in developing drugs for AD therapy. Recent clinical trials have shown that targets other than amyloid need to be found in order to create an effective therapeutic solution for the treatment of AD [40]. A large mass of data indicates that the disruption of Са^2+^ homeostasis in neurons plays a significant role in AD pathogenesis. The data in favor of the calcium hypothesis of AD development have been actively discussed in recent years [41]. This hypothesis is reviewed below. One of the key connections between AD pathogenesis and Ca^2+^ is based on data which state that Aβ oligomers can form Ca^2+^–permeable channels in membranes [42]. The ability of Aβ oligomers to associate with membranes is enhanced if the membrane is treated by phosphatidylserine (PtdS) [43], which occurs naturally in cells experiencing a deficit of energy. Age–related changes in the mitochondria can increase the amount of surface PtdS in neurons and thus facilitate the Aβ–mediated formation of pores, the uptake of Ca^2+^, and cell death (Fig. 2). In fact, neurons with decreased levels of cytosol ATP and increased levels of PtdS are especially sensitive to Aβ–mediated toxicity [44]. The ability of Aβ–oligomers to form Ca^2+^–permeable channels in the neuron plasma membrane is in agreement with the results of the most recent experiments on * in vivo *measurements of intracellular Ca^2+^ concentrations on transgenic APP mice [45]. The authors of this study demonstrated that the quiescent–state Ca^2+^ levels in approximately 35% of neuronal axons located in close proximity to amyloid plaques were reliably higher than in control cells. The most likely explanation for this fact is that the local concentration of Aβ–oligomers in the regions adjacent to the plaque causes the formation of Ca^2+^–permeable ion channels in the plasma membrane of neurons. Axons with increased Ca^2+^ levels lose their spikes and exhibit defective morphology [45]. The morphological changes in these axons can be alleviated by the activity of the calcineurin inhibitor FK–506 [45]. Based on this fact, we can hypothesize that calcineurin plays an important role in the pathological response of neurons to an increase in the level of Ca^2+^. Together with the direct effects on the permeability of the plasma membrane for Ca^2+^ ions, Aβ oligomers also affect the neuronal Ca^2+^ homeostasis via the modulation of the NMDA–receptor [46, 47] (Fig. 2), AMPA–receptor [48], and P/Q–type VGCC activity [49].

Another important relationship between Ca^2+^ signaling and AD was discovered from the fact that various mutations of presenilins found in HAD cases cause the deregulation of Ca^2+^ signaling. Initially, the connection between presenilins and Ca^2+^ signaling was discovered in a report that observed that fibroblasts from patients with HAD released abnormally high amounts of Ca^2+^ in response to the effect of InsP_3_ [50]. Similar data have been obtained in experiments on cells expressing mutant presenilins characterstic of AD [51], as well as on murine cortical neurons expressing mutant forms of presenilins characterstic of HAD [52, 53]. In order to explain these results, researchers hypothesized that mutant forms of presenilins affected the depot–controlled uptake of Ca^2+^ [54, 55], increase the activity and/or expression of intracellular Ca^2+^ ion–channels such as RyanR [53, 56, 57] and InsP_3_R [58, 59], or modulate the function of the Ca^2+^pump SERCA in the ER [60]. Research done in our laboratory demonstrates that presenilins by themselves can work as channels for the draining of Ca^2+^ from the ER and that numerous mutations of presenilins associated with HAD lead to the overstocking of the ER with Ca^2+^ and the excessive release of Ca^2+^ from the ER [61, 62]. Despite some differences in the details of the proposed mechanisms, most of the reviewed studies are in agreement with the idea that various mutations of presenilins associated with HAD lead to the excessive release of Ca^2+^ from the ER via InsP_3_R and RyanR. There are several effects from releasing Ca^2+^ through the Aβ channels and excessively releasing Ca^2+^ from the ER, which are especially toxic. As was said earlier, an increased level of cytosolic Ca^2+^ can activate calcineurin and cause atrophy [45] (Fig. 2). An excessively high level of Ca^2+^ also activates calpains, which destroy signaling enzymes involved in the processes of learning and memory [25, 63] (Fig. 2). Aged neurons become sensitive to the toxicity of cytosolic Ca^2+^ since aged cells have lower cytosolic buffering capacity.

In fact, an evident correlation has been found between the lowered expression of Са–binding proteins in the region of the *dentate gyrus* of the hippocampus and the emergence of cognitive disorders associated with AD [64]. Abnormally high cytosolic Ca^2+^ signals can cause the excessive uptake of Ca^2+^ into the mitochondria and lead to cell apoptosis (Fig. 2). The known positive effects of nonsteroid anti–inflammatory drugs can be explained by their ability to lower the mitochondrial uptake of Ca^2+^ [65].

In conclusion, we must note that a large mass of experimental data demonstrate excessive levels of Ca^2+^ in the neuronal cytosol as an effect of the Aβ oligomer accumulation or of the expression of mutant presenilins characteristic of HAD. Further proof of the connection between Ca^2+^ signaling and AD was obtained from a recent study which demonstrated that a mutation in the new Ca^2+^–uptake channel CALHM1 can increase the risk of late–onset AD [66] (however, see also [67]). The proposed model (Fig. 2) offers a whole range of potential drug targets for AD therapy. The Aβ–based Ca^2+^ channels by themselves are very promising drug targets [68]. Thus, the US Food and Drug Administration has already approved memantin, which is a noncompetitive inhibitor of the NMDA–receptor as a therapeutic drug for AD (Table 1). There is the possibility of developing even more specific inhibitors for the NMDA–receptor, such as nitromemantins [69]. Recently, Evotec Inc has developed potential AD drugs based on a specific antagonism of NR2B receptors : EVT101 and EVT103 (Table 2). An L–type VGCC inhibitor MEM–1003 (Memory Pharmaceuticals) has successfully passed second stage clinical trials ( Table 2). Other potential and mostly unstudied targets for AD therapy include intracellular Ca^2+^ channels (RyanR and InsP_3_R), the SERCA pump, calcineurin, and the mitochondrial Ca^2+^ regulation system.

The presented data constitute a new view on the therapy of neurodegenerative pathologies. Our proposed Ca^2+^ hypothesis creates a basis for the development of a new class of drugs.

## Ca^2+^ Signaling: Current Perspectives for Therapeutic Applications

**Mitochondrial stabilizers and antidepressants.** Ketasyn, Creatine, coenzyme Q10 (CoQ10), and MitoQ have all passed clinical trials for the therapy of AD and HD. Since mitochondria play a key role in the pathogenesis of these diseases [70], these clinical trials were expected to yield some positive results. However, mitochondria are involved in the pathological process at a relatively late stage, so the effect of these drugs can be expected to be limited. In fact, according to reports on this type of drugs, only modest therapeutic effects have been reported in the treatment of neurodegenerative disorders [70].

**Dimebon**. Dimebon (Medivation Inc) showed promising results (based on cognitive tests performed on patients) in the second phase of AD–therapy clinical trials [71]. Dimebon also passed the second stage clinical trials for HD therapy and demonstrated a weak effect on the brain activity of patients (Kieburtz *at al*, 2010 *Arch Neurology*, in press)

Dimebon is a well known antihistaminic drug used throughout the world and in Russia which, according to reports, had a neuroprotective effect when used in picomolar concentrations via a new effect on the mitochondria [72]. However, our studies on a culture of medium spiny neurons from the striatum showed a reliable neuroprotective effect of Dimebon only at 50 µМ concentrations [73]. We concluded that the cognitive effect of Dimebon observed in clinical trials for the treatment of AD [71] was probably caused by the ability of this drug to inhibit α–adrenergic, histamine, and serotonine high–affinity receptors [73]. In March 2010, the third stage clinical trials of Dimebon as a therapy for AD were completed and deemed to be a complete failure (http://www.alzforum.org/new/detail.asp?id=2387). Currently, it is unclear whether Dimebon will be studied further as a treatment for AD and HD.

**Antagonists of the NMDA–receptor**. Memantine is noncompetitive antagonist of the NMDA–receptor which has been approved by the FDA for the treatment of AD. Memantine is also in a clinical trial for the treatment of HD. NR 2B–specific antagonists EVT101 and EVT103 (Evotec Inc) have been developed for the treatment of AD and they are expected to be tested in second stage clinical trials soon. The same drugs are also promising therapeutic compounds for the treatment of HD.

**Riluzole**. An antiglutamate agent which has been approved by the FDA for the treatment of ALS, Riluzole has also competed third–stage clinical trials for the treatment of HD; however, it did not exhibit any reliable positive effect on the motor measurements performed on patients [19].

**Antagonists of L–type VGCC**. An “CNS–optimized” inhibitor of L–type VGCC MEM–1003 (Memory Pharmaceuticals) showed a moderate positive effect in second stage clinical trials on AD patients.

In conclusion, we must acknowledge that research in new directions of brain studies using modern molecular–biological and electrophysiological approaches will inevitably lead to an elucidation of the mechanisms behind highly effective informational flow and will also help discover approaches for treating neurodegeneration.

## ABBREVIATIONS

ER: endoplasmic reticulum
MCU: mitochondrial Ca^2+^ uniporter
AD: Alzheimer’s disease
PD: Parkinson’s disease
ALS: amyotrophic lateral sclerosis
HD: Huntington’s disease
SCA: spinocerbellar ataxias
Htt: huntingtin protein
NMDAR: N–methyl–D–aspartate receptors
MSN: medium spiny neurons
TBZ: tetrabenazine
HAD: heritable Alzheimer’s disease
PtdS: phosphatidyl serine
InsP3R1: type 1 inositol (1,4,5)–trisphosphate receptor
mPTP: mitochondrial permeability transition pore
